# First Molecular Identification of *Entamoeba* spp. in Sheep, Beef Cattle, and Dairy Cattle in Shanxi Province, North China

**DOI:** 10.3390/vetsci12010019

**Published:** 2025-01-04

**Authors:** Ze-Xuan Wu, Han-Dan Xiao, Yuan-Hui He, Shi-Bo Huang, Jing Li, Yu Kang, Wen-Bin Zheng, Xing-Quan Zhu

**Affiliations:** 1 Laboratory of Parasitic Diseases, College of Veterinary Medicine, Shanxi Agricultural University, Jinzhong 030801, China; wuzexuan0602@163.com (Z.-X.W.); 15166873600@163.com (H.-D.X.); sxhyh2024@163.com (Y.-H.H.); h17516211643@126.com (S.-B.H.); lijing1127x@163.com (J.L.); yukang2024@126.com (Y.K.); 2 The Yunnan Key Laboratory of Veterinary Etiological Biology, College of Veterinary Medicine, Yunnan Agricultural University, Kunming 650201, China

**Keywords:** beef cattle, dairy cattle, *Entamoeba* spp., prevalence, Shanxi province, sheep

## Abstract

*Entamoeba* spp. are common zoonotic intestinal protozoa, which can lead to serious intestinal diseases in both humans and animals through fecal–oral transmission, leading to significant economic losses and public health challenges. To reveal the prevalence of *Entamoeba* in sheep and cattle in Shanxi Province, North China, fecal samples were collected from 311 sheep, 392 dairy cattle, and 393 beef cattle from three representative counties in the northern, central, and southern regions of Shanxi Province. DNA was extracted from the fecal samples and amplified by PCR with primers targeting the nuclear small subunit ribosomal RNA (SSU rRNA) gene of *Entamoeba* spp., followed by the sequencing of the positive products. The overall infection rates of *Entamoeba* were 51.5% (160/311), 82.9% (325/392), and 79.1% (311/393) in sheep, dairy cattle, and beef cattle, respectively. Statistical analysis showed a significant correlation between the infection rate of *Entamoeba* and the location factor in sheep, dairy cattle, and beef cattle (*p* < 0.001). According to the obtained SSU rRNA sequences, several *Entamoeba* species, namely *Entamoeba bovis*, *Entamoeba* Ribosomal Lineage (RL) 2, *Entamoeba* RL4, and *Entamoeba* RL8, were identified. This study represents the first molecular survey of *Entamoeba* prevalence in sheep, beef cattle, and dairy cattle in Shanxi Province. The findings extend the geographical distribution of *Entamoeba* spp. and provide valuable scientific data for the prevention and control of amoebiasis in Shanxi Province.

## Simple Summary

The infection of animals and humans with *Entamoeba* spp. causes significant economic losses to the livestock industry and threatens public health. Animals are potential reservoirs for human infection with *Entamoeba* spp. Hence, investigations of *Entamoeba* infections in livestock are important for better control of human infection with *Entamoeba* spp. However, to date, infection of *Entamoeba* spp. in sheep and cattle in Shanxi Province remains unknown. In the present study, fecal samples were collected from 311 sheep, 392 dairy cattle, and 393 beef cattle from three representative counties in the northern, central, and southern regions of Shanxi Province and were investigated for *Entamoeba* spp. by PCR amplification and sequencing. The results showed that the overall infection rates of *Entamoeba* were 51.5% (160/311), 82.9% (325/392), and 79.1% (311/393) in sheep, dairy cattle, and beef cattle, respectively; and several *Entamoeba* species were identified, including *Entamoeba bovis*, *Entamoeba* Ribosomal Lineage (RL) 2, *Entamoeba* RL4, and *Entamoeba* RL8. The present study reports the occurrence and prevalence of *Entamoeba* spp. for the first time in sheep and cattle in Shanxi Province, which extended the geographical distribution of *Entamoeba* spp.

## 1. Introduction

*Entamoeba* spp. are zoonotic protozoan parasites that inhabit the intestine and other organs of their hosts and are widely distributed in the natural environment [1]. Humans and animals are primarily infected with *Entamoeba* through the ingestion of mature cysts via the fecal–oral transmission route [1,2]. The genus *Entamoeba* includes several species capable of infecting humans, including *Entamoeba histolytica*, *E. dispar*, *E. moshkovskii*, *E. coli*, *E. polecki*, and *E. hartmanni* [3,4]. Of these, *E. histolytica* is recognized as the only pathogenic species of *Entamoeba* that causes amebic dysentery and other far-ranging diseases, including self-limiting colitis, invasive colitis, extra-intestinal infection, and invasive organ abscesses [5,6,7].

Amebiasis ranks as the third leading cause of parasitic mortality worldwide, following malaria and schistosomiasis [8,9]. It is estimated that approximately 500 million of the global population is infected with *Entamoeba*, with around 40,000–100,000 deaths annually due to amebiasis [1]. An epidemiological survey conducted in a small town in Ethiopia showed that 13.17% of children were infected with *E. histolytica* [10,11], highlighting its significant impact on child health in developing countries [1,12]. In addition to being identified in humans, *Entamoeba* infection has been reported in various animals, including pigs, alpaca, deer, and yaks, across multiple regions such as Australia, China, Costa Rica, Iceland, Japan, Libya, Sweden, Uganda, and the United Kingdom [13,14,15,16,17,18].

So far, several *Entamoeba* species/genotypes have been reported in ruminants, such as *E. bovis* and *Entamoeba* Ribosomal Lineage (RL) 1, *Entamoeba* RL2, and *Entamoeba* RL4 [19]. However, the prevalence and genetic characteristics of *Entamoeba* infection in sheep, dairy cattle, and beef cattle remain limited in China. Thus, the present study aimed to investigate the prevalence of *Entamoeba* in sheep, dairy cattle, and beef cattle in Shanxi Province, North China, and to identify the species/genotypes through PCR amplification and sequencing of the nuclear small subunit ribosomal RNA (SSU rRNA) sequences [20]. The findings are intended to provide essential data for the prevention and control of amoebiasis in Shanxi province.

## 2. Materials and Methods

### 2.1. Sample Collection

**Figure 1:**
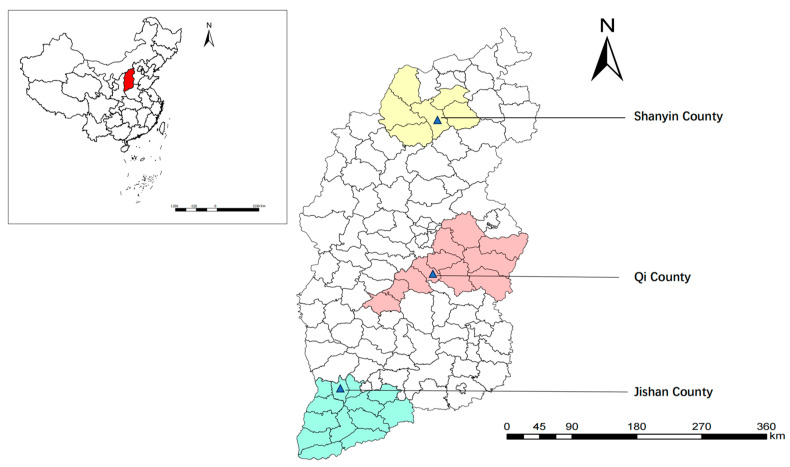
Sampling sites of sheep and cattle feces in Shanxi province, North China. The map was generated by ArcGIS10.8 using data from the Resource and Environmental Science and Data Center of the Chinese Academy of Sciences.

From May to November 2020, 311 fresh fecal samples from sheep (97 from Qi County, 108 from Jishan County, and 106 from Shanyin County), 392 fecal samples from dairy cattle (137 from Qi County, 49 from Jishan County, and 206 from Shanyin County), and 393 fecal samples from beef cattle (174 from Qi County and 219 from Jishan County) were collected from three representative counties of Shanxi province (Figure 1), respectively. The fecal samples were carefully separated using sterile polyethylene gloves to prevent contamination, and relevant sample information (e.g., region, sex, age, and host species/breed) was recorded. Each sample weighed between 10 g and 20 g. The collected samples were transported to the Laboratory of Parasitic Diseases, College of Veterinary Medicine, Shanxi Agricultural University, and stored at −20 °C for further analysis [21,22].

### 2.2. DNA Extraction and PCR Amplification

The genomic DNA was extracted from each of the mixed fecal samples (approximately 200 mg) using the E.Z.N.A.^®^ Stool DNA Kit (Omega Bio-Tek Inc., Norcross, GA, USA) following the manufacturer’s instructions and was stored at −20 °C. The SSU rRNA gene of *Entamoeba* spp. was amplified by specific primers, EntboF2 (5′-TAAGAGGAACAATTGGGGTGAT-3′) and R3 (5′-AGGAATTCCTCGTTCAAAACAAA-3′), as described in a previous study [16]. The PCR reaction mixture consisted of 17.8 µL of ddH_2_O, 2.5 µL of 10× buffer (Mg^2+^ plus), 2 µL of dNTP (2.5 mM), 0.2 µL of Ex *Taq*, 1.5 µL of template DNA, and 0.5 µL of upstream and downstream primers. The PCR reaction conditions involved an initial pre-denaturation at 95 °C for 5 min, followed by 35 cycles of denaturation at 95 °C for 30 s, annealing at 55 °C for 30 s, and extension at 72 °C for 1 min, concluding with a final extension at 72 °C for 5 min, and cooling to 16 °C. Subsequently, the PCR products were examined through electrophoresis on a 1.5% agarose gel containing ethidium bromide and were visualized under ultraviolet light.

### 2.3. Sequence and Phylogenetic Analyses

Positive PCR amplicons (approximately 800 bp) were sent to Sangon Biotech Company (Shanghai, China) for sequencing. The species/genotypes of *Entamoeba* were determined by aligning and comparing the obtained sequences with known sequences available in GenBank using BLAST (http://www.ncbi.nlm.nih.gov/BLAST/, accessed on 5 July 2024). The phylogenetic relationship of species/genotypes was established by the neighbor-joining (NJ) method using the Kimura-2-parameter model in MEGA 7 software. The bootstrap value was set to 1000 times to evaluate the robustness of clusters.

### 2.4. Statistical Analysis

In this study, statistical analysis was performed using SPSS 26.0 (IBM, Chicago, IL, USA). The relationships between factors (e.g., region, age, and sex) and the infection rate of *Entamoeba* was assessed using the Chi-square test (χ2). The odds ratios (ORs) and corresponding 95% confidence intervals (CIs) were calculated, with a *p*-value < 0.05 being considered statistically significant.

## 3. Results

### 3.1. Prevalence of Entamoeba spp. in Sheep

In this study, 160 of 311 sheep fecal samples tested positive for *Entamoeba*, resulting in an overall infection rate of 51.5% (160/311, 95% CI: 45.9–57.0) (Table 1). The highest infection rate was observed in sheep in Qi County (72.2%, 70/97, 95% CI: 63.2–81.1), followed by Jishan County (44.3%, 47/106, 95% CI: 34.9–53.8) and Shanyin County (39.8%, 43/108, 95% CI: 30.6–49.0), with significant regional differences (*p* < 0.001). In addition, lamb (≤6 months) had a significantly higher infection rate (75.7%, 53/70, 95% CI: 65.7–85.8) than older sheep (>6 months) (44.4%, 107/241, 95% CI: 38.1–50.7), showing a strong correlation between *Entamoeba* infection and age in sheep (*p* < 0.001).

### 3.2. Prevalence of Entamoeba spp. in Dairy Cattle

In dairy cattle, the prevalence of *Entamoeba* spp. was 82.9% (325/392, 95% CI: 79.2–86.6) in this study (Table 2). The highest prevalence was observed in dairy cattle in Jishan County (95.9%, 47/49, 95% CI: 90.4–100.0), followed by Qi County (89.1%, 122/137, 95% CI: 83.8–94.3) and Shanyin County (75.7%, 156/206, 95% CI: 69.9–81.6). Furthermore, the infection rates of *Entamoeba* were 82.7% (268/324, 95% CI: 78.6–86.8) in female cattle and 83.8% (57/68, 95% CI: 75.1–92.6) in male cattle, showing no significant gender difference (*p* > 0.05). Among the age groups, the infection rates of *Entamoeba* were 83.3% (180/216, 95% CI: 78.4–88.3) in adult dairy cattle (>18 months) and 82.4% (145/176, 95% CI: 76.8–88.0) in young dairy cattle (≤18 months), with no significant difference (*p* > 0.05).

### 3.3. Prevalence of Entamoeba spp. in Beef Cattle

The overall prevalence of *Entamoeba* spp. in beef cattle from Qi County and Jishan County was 79.1% (311/393, 95% CI: 75.1–83.2) (Table 3). Notably, beef cattle in Jishan County had a significantly higher *Entamoeba* prevalence (85.8%, 188/219, 95% CI: 81.2–90.5) than those in Qi County (70.7%, 123/174, 95% CI: 63.9–77.5) (*p* < 0.001). No significant difference was found in the prevalence of *Entamoeba* between male beef cattle (81.6%, 151/185, 95% CI: 76.0–87.2) and female beef cattle (76.9%, 160/208, 95% CI: 71.2–82.6) (*p* > 0.05). However, the prevalence of *Entamoeba* in young beef cattle (≤12 months) (81.8%, 243/297, 95% CI: 77.4–86.2) was higher than that in adult beef cattle (>12 months) (70.8%, 68/96, 95% CI: 61.7–79.9), with a statistically significant difference (*p* < 0.05).

### 3.4. Sequence Alignment and Phylogenetic Analysis of Entamoeba

**Figure 2:**
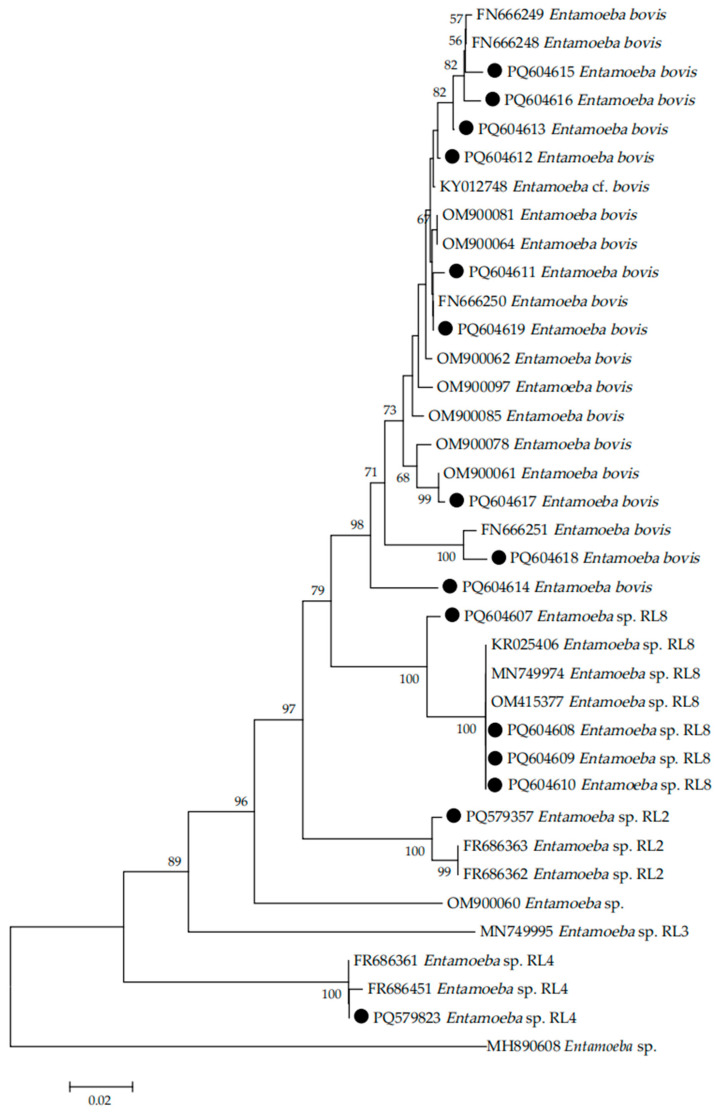
Phylogenetic relationships of *Entamoeba* spp. based on the sequences of the small subunit ribosomal RNA (SSU rRNA) using the neighbor-joining (NJ) method. Representative *Entamoeba* SSU rRNA sequences obtained in this study are labeled with black circles (●). The bootstrap value is shown when >50%.

The samples with successful PCR amplification (approximately 800 bp) were sequenced, and phylogenetic analysis was performed. In the present study, 15 distinct sequences were identified (GenBank accession numbers PQ579357, PQ579823, and PQ604607–PQ604619) through sequencing analysis. The genetic distance between different species/genotypes was analyzed to determine their relationship. The results showed that the *Entamoeba* species/genotype infecting sheep, dairy cattle, and beef cattle in Shanxi Province were *E. bovis*, *Entamoeba* RL2, *Entamoeba* RL4, and *Entamoeba* RL8 (Figure 2).

## 4. Discussion

*Entamoeba* spp. are among the most prevalent intestinal parasites in humans and large mammals, and they often parasitize ruminant animals such as cattle and sheep [8]. The prevalence of *Entamoeba* infection in animals has been documented domestically and internationally [23,24]. However, at present, research on enteric *Entamoeba* infection in even-toed ungulates in China is limited, particularly in Shanxi Province. To the best of our knowledge, the present study is the first to investigate *Entamoeba* spp. infection in sheep, dairy cattle, and beef cattle in Shanxi Province, North China using a molecular approach.

Previous studies have reported the infection of *Entamoeba* in ruminants in China by PCR technology. The infection rate of *Entamoeba* in sika deer in Anhui Province, China, was 31.5% (106/336); in Qinghai Province, China, the overall positive rate of *Entamoeba* among yaks was 36.32% (373/1027); in Shanxi Province, China, the overall positive rate of *Entamoeba* among alpacas was 18.03% (66/366); and it was 100% (57/57) in farm animals on the Qinghai-Tibetan Plateau of China [14,23,25,26]. The differences in the prevalence of *Entamoeba* may be due to several factors, such as geographical locations, detection methods, management patterns, and sampling sizes. Our study revealed a high *Entamoeba* prevalence of 82.9% (325/392) in dairy cattle and 79.1% (311/393) in beef cattle.

In the present study, the prevalence of *Entamoeba* infection in sheep, dairy cattle, and beef cattle was significantly different (*p* < 0.001) in different regions. In sheep, the highest prevalence of *Entamoeba* was detected in Qi County (72.2%), followed by Jishan County (44.3%) and Shanyin County (39.8%). The highest *Entamoeba* prevalence in cattle was observed in Jishan County, where the prevalence in dairy cattle (95.9%) and beef cattle (85.8%) was notably higher than that in Qi County (89.1% in dairy cattle and 70.7% in beef cattle) and Shanyin County (75.7% in dairy cattle). According to these findings, the higher prevalence of *Entamoeba* in sheep in Qi County might be related to local climate, farm hygiene conditions, feeding management patterns, and sample sizes [13]. However, the elevated positivity rate in dairy cattle and beef cattle in Jishan County could be associated with the regional latitude [21,27].

In terms of sex groups, the prevalence of *Entamoeba* did not exhibit significant differences between male dairy cattle (83.8%) and female dairy cattle (82.7%) (*p* > 0.05) or between male beef cattle (81.6%) and female beef cattle (76.9%) (*p* > 0.05). These findings suggest that sex does not play a significant role in the prevalence of *Entamoeba* in either dairy cattle or beef cattle. Our study revealed significant differences in the prevalence of *Entamoeba* in different age groups in sheep (*p* < 0.001), with lambs exhibiting a higher prevalence than older sheep. This suggests that age may influence the susceptibility to *Entamoeba* infection in sheep, potentially due to factors such as immune system development [25]. Additionally, a significant difference was observed in the prevalence of *Entamoeba* in beef cattle (*p* = 0.021), whereas no significant difference was detected in the prevalence of *Entamoeba* in dairy cattle (*p* > 0.05). These findings suggest that while sex does not appear to influence *Entamoeba* prevalence in either dairy or beef cattle, age-related differences in sheep and certain variations between beef cattle and dairy cattle indicate that factors other than sex, such as age and possibly breed, may play a more significant role in *Entamoeba* infection dynamics [28]. The high prevalence of *Entamoeba* observed in this study could be attributed to insufficient awareness and lack of preventive measures among managers of these farms, which may exacerbate the spread of these parasites.

In this study, phylogenetic analysis revealed the presence of *E. bovis*, *Entamoeba* RL2, *Entamoeba* RL4, and *Entamoeba* RL8 in the sampled ruminant populations, which extends our understanding of the genetic diversity and host range of *Entamoeba* spp. in Shanxi Province. The term “ribosomal lineage” (RL) refers to newly discovered *Entamoeba* 18S rRNA sequences that diverge by more than 5% from known species [29]. RLs are used to classify organisms with phylogenetic branches distinct from previously described *Entamoeba* species, and 11 RLs have been identified [19,30]. The phylogenetic evolutionary analysis suggests that *E. bovis* can be isolated from ruminant hosts other than cattle [31,32]. To our knowledge, this study represents the first species-specific identification of *Entamoeba* in sheep, dairy cattle, and beef cattle in Shanxi Province, and further studies are warranted to enhance the better understanding of *Entamoeba* infection in ruminants in Shanxi province by sampling larger numbers of animals and more geographical locations.

## 5. Conclusions

This study represents the first on the occurrence and prevalence of *Entamoeba* spp. in sheep, dairy cattle, and beef cattle in Shanxi Province. *Entamoeba* prevalence was 51.5%, 82.9%, and 79.1%, respectively, in these ruminants. According to the obtained SSU rRNA sequences, the identified *Entamoeba* included *E. bovis*, *Entamoeba* RL2, *Entamoeba* RL4, and *Entamoeba* RL8, extending our understanding of the geographical distribution of *Entamoeba* in ruminants. Furthermore, these findings underscore the necessity for ongoing research to fully comprehend the prevalence and impact of *Entamoeba* spp. in different regions and host species.

## Figures and Tables

**Table 1:** Factors associated with prevalence of *Entamoeba* spp. in sheep.

Factor	Category (County)	No. Tested	No. Positive	Prevalence % (95% CI)	Odds Ratio (95% CI)	*p*-Value
Region	Shanyin	108	43	39.8 (30.6–49.0)	1	<0.001
	Qi	97	70	72.2 (63.2–81.1)	3.9 (2.2–7.1)	
	Jishan	106	47	44.3 (34.9–53.8)	1.2 (0.7–2.1)	
Age	M > 6	241	107	44.4 (38.1–50.7)	1	<0.001
	M ≤ 6	70	53	75.7 (65.7–85.8)	3.9 (2.1–7.1)	
Total		311	160	51.5 (45.9–57.0)		

**Table 2:** Factors associated with prevalence of *Entamoeba* spp. in dairy cattle.

Factor	Category (County)	No. Tested	No. Positive	Prevalence % (95% CI)	Odds Ratio (95% CI)	*p*-Value
Region	Shanyin	206	156	75.7 (69.9–81.6)	1	<0.001
	Qi	137	122	89.1 (83.8–94.3)	2.6 (1.4–4.9)	
	Jishan	49	47	95.9 (90.4–100.0)	7.5 (1.8–32.1)	
Sex	Male	68	57	83.8 (75.1–92.6)	1.1 (0.5–2.2)	0.825
	Female	324	268	82.7 (78.6–86.8)	1	
Age	M > 18	216	180	83.3 (78.4–88.3)	1.1 (0.6–1.8)	0.804
	M ≤ 18	176	145	82.4 (76.8–88.0)	1	
Total		392	325	82.9 (79.2–86.6)		

**Table 3:** Factors associated with prevalence of *Entamoeba* spp. in beef cattle.

Factor	Category (County)	No. Tested	No. Positive	Prevalence % (95% CI)	Odds Ratio (95% CI)	*p*-Value
Region	Qi	174	123	70.7 (63.9–77.5)	1	<0.001
	Jishan	219	188	85.8 (81.2–90.5)	2.5 (1.5–4.2)	
Sex	Male	185	151	81.6 (76.0–87.2)	1.3 (0.8–2.1)	0.253
	Female	208	160	76.9 (71.2–82.6)	1	
Age	M > 12	96	68	70.8 (61.7–79.9)	1.9 (1.1–3.1)	0.021
	M ≤ 12	297	243	81.8 (77.4–86.2)	1	
Total		393	311	79.1 (75.1–83.2)		

## Data Availability

The datasets supporting the results of this article have been submitted to GenBank, and the accession numbers are shown in the article.
